# Strategically positioning cooperators can facilitate the contagion of cooperation

**DOI:** 10.1038/s41598-020-80770-8

**Published:** 2021-01-13

**Authors:** Guoli Yang, Matteo Cavaliere, Cheng Zhu, Matjaž Perc

**Affiliations:** 1 Unit 66136, Beijing, 100042 China; 2 Science and Technology on Information Systems Engineering Laboratory, National University of Defense Technology, Changsha, 410073 China; 3 Department of Computing and Mathematics, Manchester Metropolitan University, Manchester, UK; 4 Faculty of Natural Sciences and Mathematics, University of Maribor, Koroška cesta 160, 2000 Maribor, Slovenia; 5 Department of Medical Research, China Medical University Hospital, China Medical University, Taichung, Taiwan; 6 Complexity Science Hub Vienna, Josefstädterstraße 39, 1080 Vienna, Austria

**Keywords:** Statistical physics, thermodynamics and nonlinear dynamics, Physics

## Abstract

The spreading of cooperation in structured population is a challenging problem which can be observed at different scales of social and biological organization. Generally, the problem is studied by evaluating the chances that few initial invading cooperators, randomly appearing in a network, can lead to the spreading of cooperation. In this paper we demonstrate that in many scenarios some cooperators are more influential than others and their initial positions can facilitate the spreading of cooperation. We investigate six different ways to add initial cooperators in a network of cheaters, based on different network-based measurements. Our research reveals that strategically positioning the initial cooperators in a population of cheaters allows to decrease the number of initial cooperators necessary to successfully seed cooperation. The strategic positioning of initial cooperators can also help to shorten the time necessary for the restoration of cooperation. The optimal ways in which the initial cooperators should be placed is, however, non-trivial in that it depends on the degree of competition, the underlying game, and the network structure. Overall, our results show that, in structured populations, few cooperators, well positioned in strategically chosen places, can spread cooperation faster and easier than a large number of cooperators that are placed badly.

## Introduction

The dynamics of various influence spreading models lies on the stochastic triggering of interactions from one state to another between connecting individuals, including independent cascade model, linear threshold model, epidemic model (such as SI, SIS, SIR), and voter models^1–3^. In order to reach the maximization of influence spreading, researchers have tried many ways to identify the influential initial spreaders, which are usually regarded as the most vital nodes in the networks. As reviewed in^4^, more than 30 methods have been proposed to characterize the *importance* of a node, including degree centrality^5^, betweenness centrality^6^, closeness centrality^7^, k-shell^8^, eigenvector centrality^9^, PageRank^10^, and HITs^11^. Based on those ranking methods, researchers can identify the nodes located in the important places, which will lead to large-scale, efficient, and in general fast, spreading.

When it comes to evolutionary populations^12–19^, individuals with different strategies interact with each other and compete for the dominance of the population, and many heuristic imitation or reaction methods have been proposed to promote the cooperation^20–23^. In particular, introducing some defective (cheating) individuals into a cooperative community, the defectors will avoid the cost of contributing to the community and can occasionally spread in the population leading to the complete collapse of cooperation^24,25^. Just like the initial spreaders in propagation networks, it is quite important to identify the most influential defectors in a population of cooperators to establish their influence on the collapse of cooperation^26^. Notably, under the framework of evolutionary game theory^27^, the identification of the most influential invaders has to consider the fact that the individuals reproduce and disappear under the effects of natural selection. In addition, there is not an ubiquitous measurement which can be applied to different conditions (such as different games, selection strength, or network topology, to name just some examples).

This issue is relevant in studying the *restoration of cooperation* in a population of cheaters. This is the process that happens when, few cooperators, introduced in a network of cheaters will spread in the network and restore cooperation.

Given a structured population (e.g., in a network) a natural question is where to add the initial cooperators and which choice facilitates the restoration of cooperation. This paper, for the first time to our knowledge, explores this question by systemically analyzing the influence of games, selections, networks, and ranking strategies on the restoration of cooperation. We study the problem on a classical model of evolutionary game model based on networks where a death-birth rule is used to model the competition between cooperators and defectors. We analyze different ways to add cooperators into a network of cheaters (using 6 standard ranking methods) and evaluate their performance in terms of the restoration of cooperation.

## Computational model

We use a model of evolutionary game theory^28^ where the players hold a game strategy and occupy the vertices of a graph; the connections between indicate the interaction between the players. The players interact with their neighbours to obtain a payoffs which depend on the player and on the neighbours strategies. The population structure (network) is fixed and the size of population is constant. Players can adopt two strategies: cooperation (*C*) and defection (*D*). In a population of *N* individuals consisting of cooperators and defectors, the total payoff of a player is the sum of the payoffs obtained from the interactions with its neighbours, namely $\pi _i = \sum _{j \in NB _i} \Pi (s_i, s_j)$. The payoff matrix can be represented by:1

Due to the differences in the configurations of neighbourhood as well as the game strategies, the payoffs obtained from the interactions can be different for the distinct players, leading to the difference in competition between the players. In general, the *fitness of a player* is given by a constant term (baseline fitness) plus the payoff obtained by interacting with the neighbours.

We define the fitness of a player as:

2 $$\begin{aligned} f_i = 1-w+w\pi _i \end{aligned}$$

where *w* is the intensity of selection (selection strength).

In this study, we use a traditional ’death-birth’ model^28^ to update the strategies present in the population. The intuition is that the strategy of a player who does well is more likely to be imitated by others (or more likely to be selected to reproduce).

**Figure 1:**
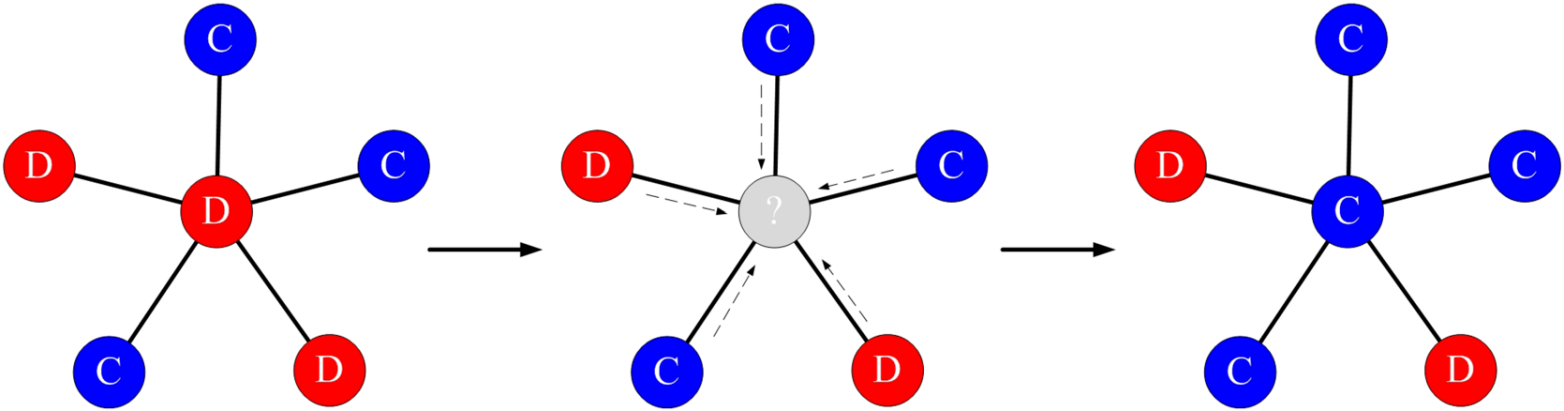
An update step of the model. Each individual occupies the vertex of graph and derives a payoff from the interactions with its neighbours. A random node is selected to die and its place is occupied by one of its neighbours with probability proportional to fitness.

An *update step* of the studied computational model works as follow: at each step, a random node *i* is selected to die, and its neighbours compete to occupy the empty site with probability proportional to their fitness. Update steps are repeated until one of the two states is reached - all nodes are cooperators or all nodes are defectors. The evolutionary dynamics can be then defined by a discrete sequence of update steps of the type shown in Fig. 1.

In the model, the competition between cooperators and defectors is highly dependent on the neighbourhood interactions and configurations. To shed light upon the influences of the positions where the initial cooperators are placed in a network of cheaters, we consider different ways to add the cooperators, according to a variety of standard *ranking strategies*. In a population composed by cheaters, we intend to pick *n* nodes as initial cooperators and evaluate whether they will survive and spread in the population, possibly leading to the restoration of cooperation.

In particular, we consider 6 popular ranking strategies which allows to explore 6 different ways to select *n* nodes as initial cooperators in a population of cheaters. The idea is to pick *n* nodes as cooperators (the so-called *top-n* nodes), in an initial network of all cheaters, and see whether they will survive and spread in the population leading to a complete restoration of cooperation. The initial density of cooperators is defined by $\rho =n/N$. Depending on the type of ranking, the initial n defectors are selected in different ways. We investigate the following rankings which describe different ways to select the *top-n nodes* (see also the Methods section)

- random ranking: the *top-n* nodes in the network are randomly chosen (this is the strategy most often used in literature)
- degree ranking: the *top-n* nodes are selected according to the ranking of degree, that is to say higher is the node degree, higher the chances that the node is selected.
- betweenness ranking: the *top-n* nodes are selected according to the ranking of betweenness (i.e., higher is the betweenness of a node, higher is the chance that the node is selected.
- k-shell ranking: the *top-n* nodes are selected according to the ranking of k-shell^8^.
- N-weighted degree ranking: the *top-n* nodes are selected based on the negative weighted degree^25^.
- P-weighted degree ranking: the *top-n* nodes are selected based on the positive weighted degree^25^.

All rankings except the random one are called *structural rankings* because they are based on some aspects of the network structure.

The performance of the different rankings (in terms of restoration of cooperation) depends the structure of network, the strength of selection, the game parameters and the density of initial cooperators. In order to gain a deep understand of those factors and their effects on the restoration of cooperation, we conduct a series of numerical simulations of the presented model. In particular, to evaluate the chances of cooperation restoration in a population full of defectors, and with different positioning of the initial cooperators, we conduct a large number of numerical simulations following this approach. At the beginning, we consider a structured population of defectors (organized on a network) with size of $N=500$, where some nodes are selected as initial cooperators according the above described ranking strategies. Based on the described evolutionary dynamics, cooperators and defectors will compete for the empty sites and we evaluate whether the cooperators survive and eventually dominate the entire population (which corresponds to the restoration of cooperation).

### Positioning the initial cooperators

**Figure 2:**
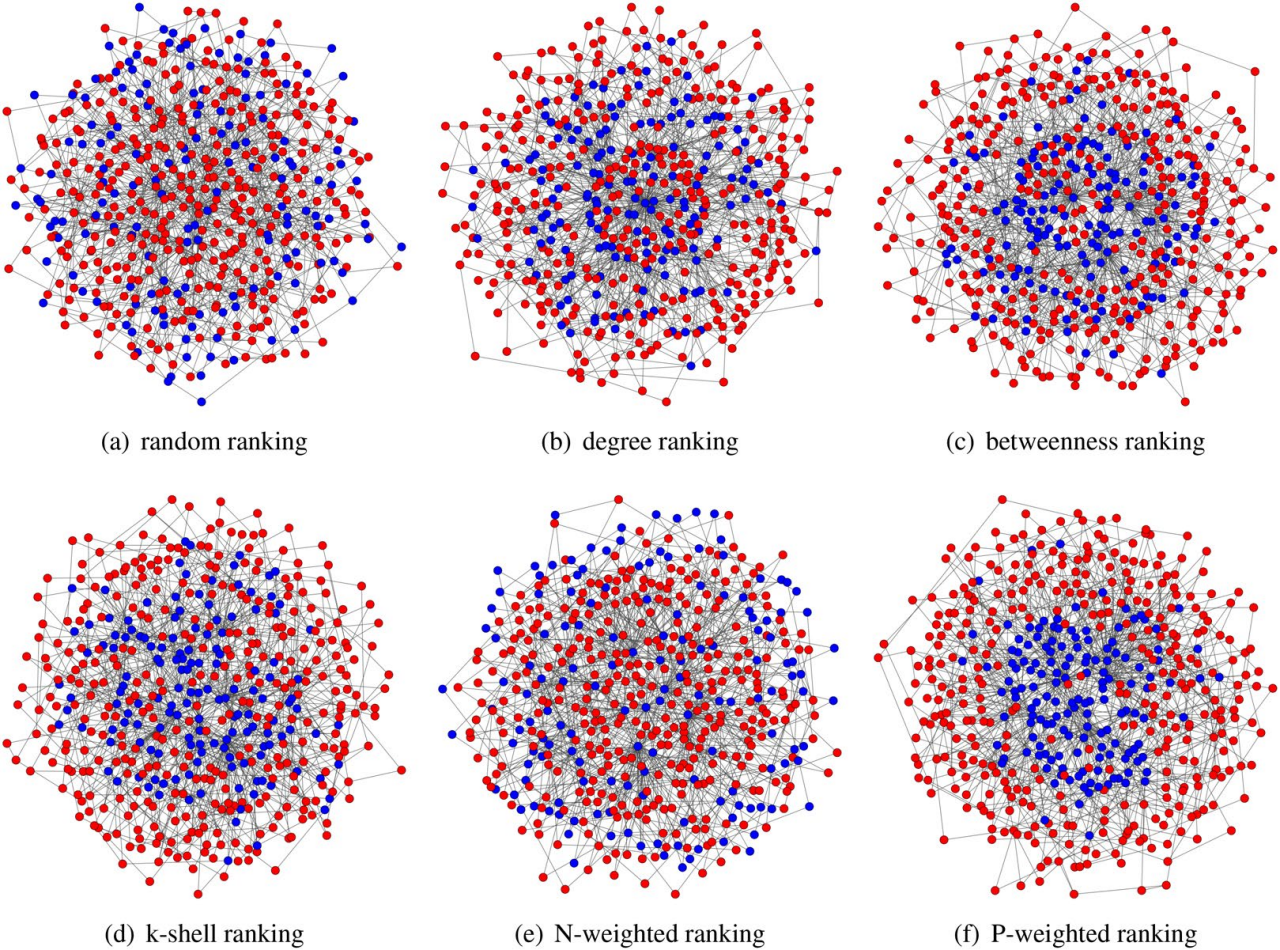
The initial positions of cooperators in a scale-free structure. The cooperators are more likely to be around the hubs when the rankings are P-weighted degree, k-shell or betweenness; however it is more likely for cooperators to be kept far away from each other when they are added using the ranking of N-weighted degree or random. We consider an initial density of $\rho = 30\%$, and cooperators are added in a network of cheaters by using the random, degree, betweenness, k-shell, N-weighted and P-weighted rankings.

First, we highlight that the positions of the initial cooperators can be very different depending on the different ranking strategies. In a scale-free network with $N=500$ nodes, the average degree is $\langle k \rangle =4$. The initial network snapshots for the different ranking strategies are illustrated in Fig. 2 when $\rho =30\%$ (i.e., $30\%$ of the nodes in the population are selected as cooperators). As we can see, the initial cooperators are more likely to get together around the hubs under the ranking of degree, k-shell, P-weighted degree etc.  , however, for the random ranking or N-weighted degree, the nodes far away from the cluster of cooperators are more likely to be selected to avoid the overlap sphere of influence.

**Figure 3:**
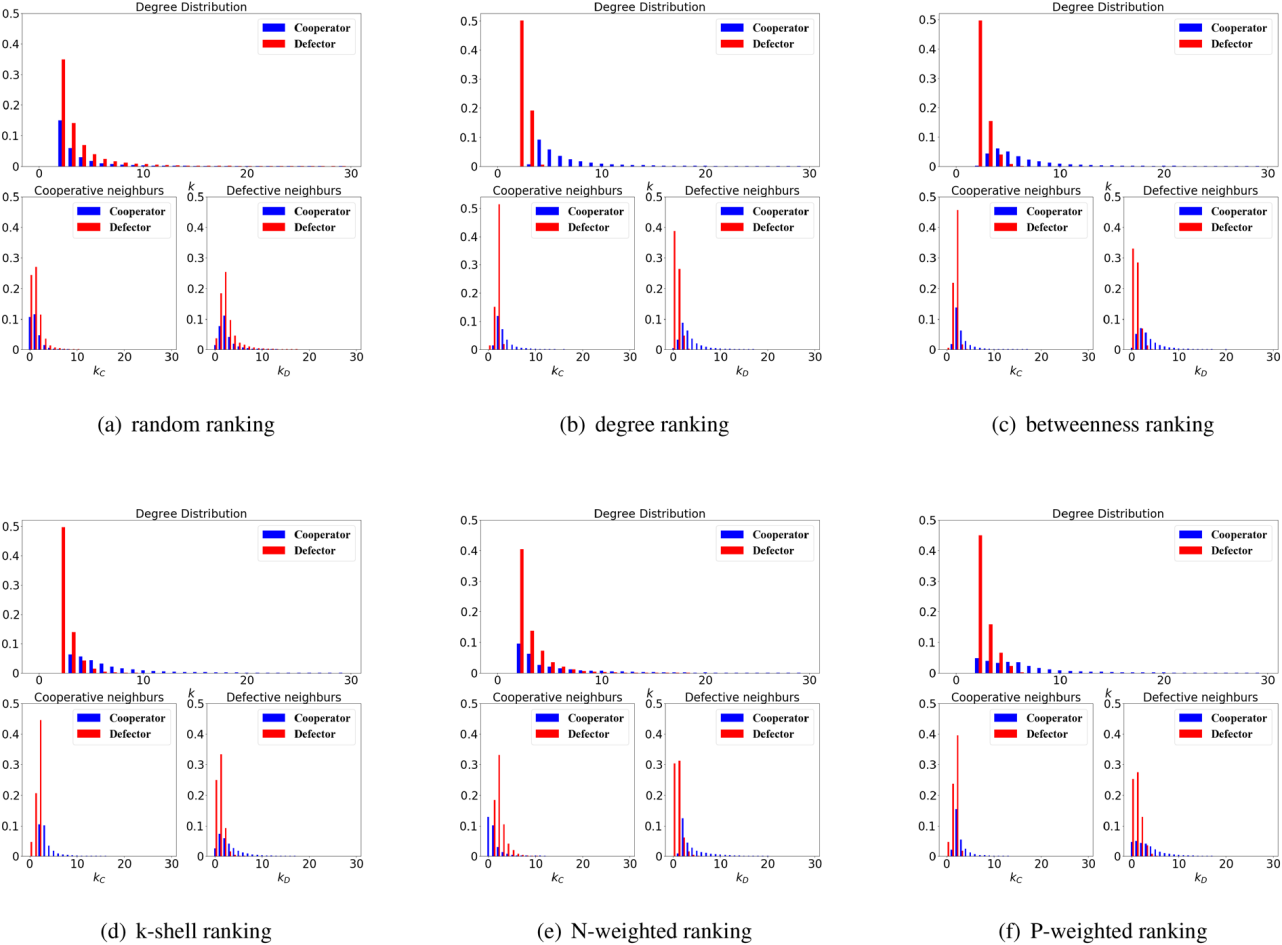
Initial degree and neighbours distributions for cooperators and defectors. The network is scale-free, with $\rho =30\%$ and the cooperators are chosen according to the ranking strategies based on random, degree, betweenness, k-shell, N-weighted degree and P-weighted degree.

To analyze in a more detailed way the positions of initial cooperators, we plot the distributions for the degree (*k*), the number of cooperative neighbours ($k_C$) and the number of cheating neighbours ($k_D$) for cooperators (blue bars) and defectors (red bars) in Fig. 3. We define the degree diversity (*dd* in short) by the variance of degree of nodes in the following way:

3 $$\begin{aligned} dd = \frac{1}{N} \sum _i (k_i-\bar{k})^2 \end{aligned}$$

As we can see, different ranking strategies will bring strong differences for the positions of the initial cooperators. In particular, for the ranking of random and N-weighted degree, the initial cooperators will keep distanced from each other and the degree distributions for cooperators and defectors are overlapping (Fig. 2); on the other hand, the cooperators selected have high degree while the defectors remain with low degree with the rankings based on degree, betweenness, k-shell.

In particular, the cooperators have more defective neighbours than the defectors with the rankings based on N-weighted degree; while, on the other hand, the defectors have more cooperative neighbours than the cooperators (Figs. 2, 3), which is consequence of the weighted degree decomposition (shown in methods).

### The restoration of cooperation

**Figure 4:**
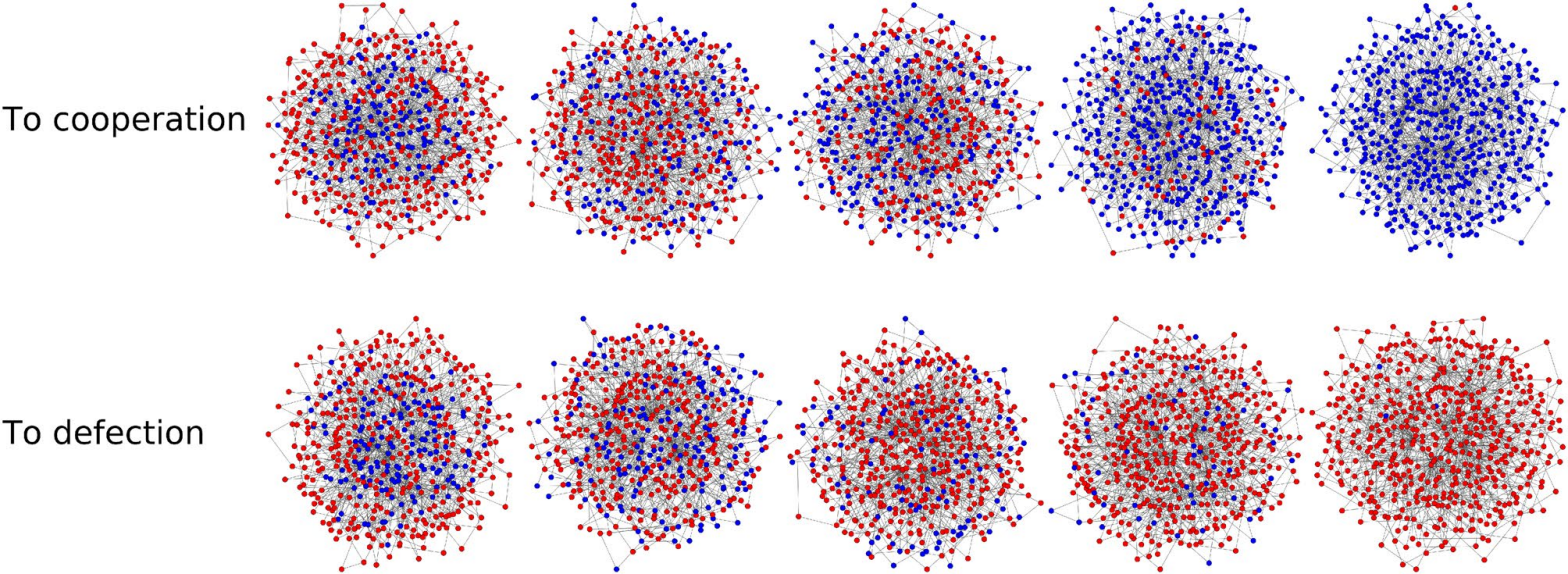
The typical trajectories of the two possible outcomes of when cooperators are introduced in a population of defectors. The initial density of cooperators is $\rho = 30\%$, and the network structure is a scale-free. The restoration of cooperation (upper panel) and the collapse of cooperation (lower panel) are shown in the case when the initial cooperators have been added using the ranking based on degree, with weak selection strength and the game is the prisoner’s dilemma.

Generally speaking, in the presented computational model, two outcomes are possible when adding cooperators in a population of cheaters: one is the restoration of cooperation (i.e., full cooperation invasion) and the other is the recovery of cheaters, i.e., when the added cooperation are eliminated from the network (the two scenarios are shown in Fig. 4). We analyze the probability of the restoration of cooperation as a way to measure the performance of the different rankings—a formal way to study which ranking (i.e., way to position the initial cooperators) facilitates the restoration of cooperation. The probability of the restoration of cooperation is simply computed as the ratio between the number of cases where the restoration of cooperation is observed and the total number of simulations. As we will see, the described computational model, the probability of restoration is highly influenced by the parameters of the games, by the selection strengths *w*, by the networks structures, by the initial density $\rho$, and by the ranking strategies used.

In this paper, we consider different games by fixing $R=1$ and $P=0$, while *S* is varied within the interval $[-1,1]$ and *T* is varied in the interval [0, 2]; in this way, different types of games can be explored, including stag-hunt game ($S\in [-1,0]$ and $T\in [0,1]$), prisoner’s dilemma ($S\in [-1,0]$ and $T\in [1,2]$), harmony game ($S\in [0,1]$ and $T\in [0,1]$) and snowdrift game ($S\in [0,1]$ and $T\in [1,2]$)^29,30^.

For the network structures, we consider a lattice, small-world^31^, random^32^ and scale-free^33^. We fix the number of nodes *N*, and the average degree $\langle k \rangle$. These types of networks have very different diversity of degree (with the special case of no diversity in the lattice). In particular, in scale-free networks, a few nodes (hubs) will have a large number of neighbours, but most nodes are only sparsely connected.

For the strength of selection, we consider $w=0.001$ (weak selection), $w=0.01$ (medium selection), and $w=0.1$ (strong selection).

For the initial density of cooperators, we study $\rho \in [0,1]$ to analyze its influence on the probability of restoration. As we expect, a bigger value of $\rho$ will lead to a larger fraction of initial cooperators, which will be more likely to form clusters of cooperators and then facilitate the restoration of cooperation. The way the initial cooperators are added, however, can dramatically affect the chances to see the spreading of cooperation and its restoration.

### Well placed cooperators can more easily spread

**Figure 5:**
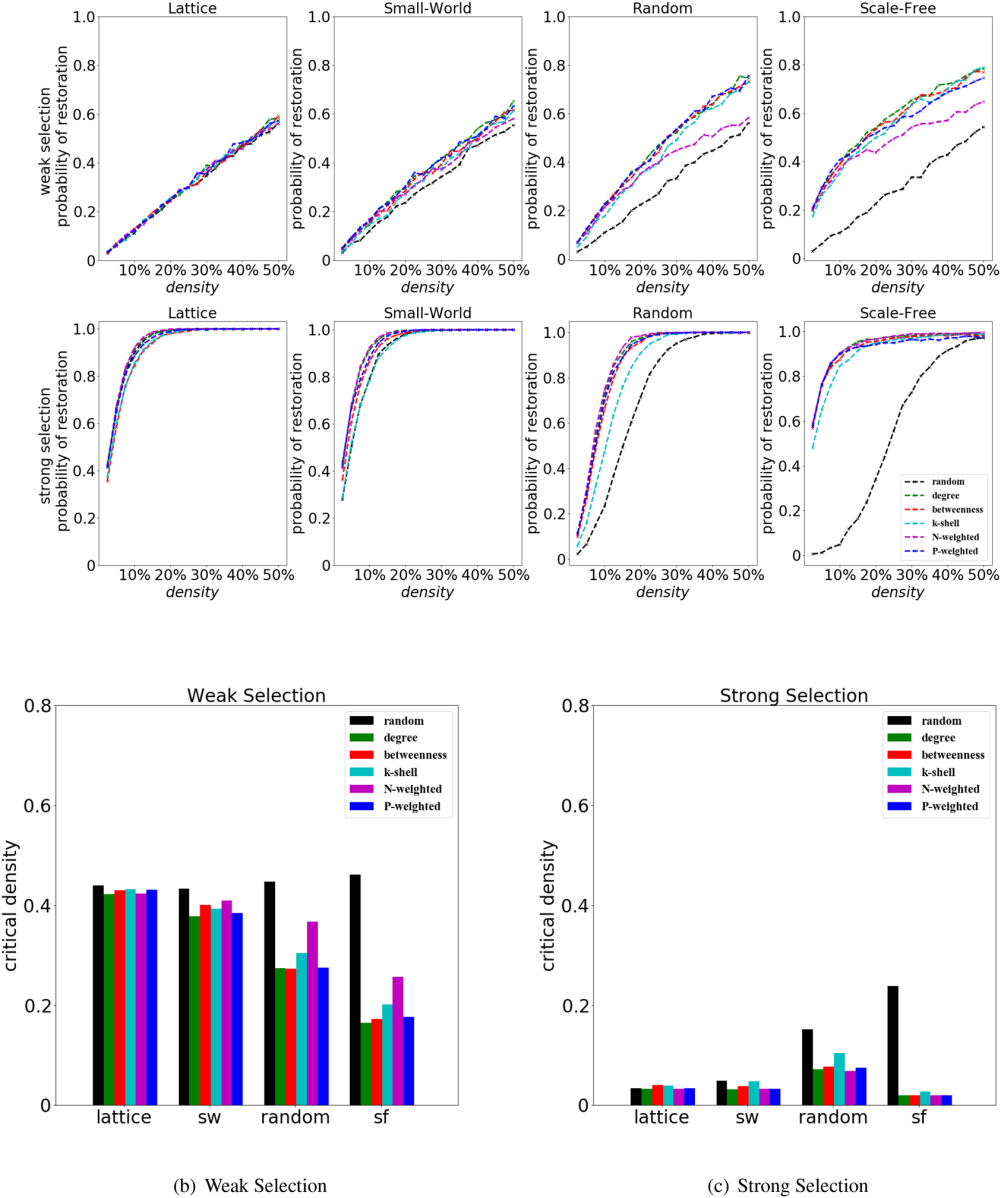
The effects of the initial cooperation density $\rho$ and positioning on the restoration of cooperation. Structural rankings will promote the restoration of cooperation with less initial cooperators than the random ranking especially when the network has a large degree diversity. For the stag-hunt game (with $S=-0.5$ and $T=0.5$), we show the probability of restoration and the critical density (i.e, initial cooperators density at which the probability of cooperation restoration reaches 0.5) in the cases of a lattice, small-world, random and scale-free networks for weak selection ($w=0.001$) strong selection ($w=0.1$).

In general, with the increase of the density of initial cooperators $\rho$, the restoration of cooperation will be enhanced. However the initial positioning of the cooperators has a big effect on the cooperation restoration. We can observe in Fig. 5 that it is possible to get high chances of cooperation restoration by either adding a large number of initial cooperators (but not placing them very strategically, e.g., using a random ranking) or a much smaller number of initial cooperators but positioned in strategic places (e.g., using appropriate structural rankings). This is due to the difference of the rankings in the selection of initial cooperators; for some rankings the initial cooperators are strategically placed in the community of defectors (such as degree ranking, k-shell ranking, weighted-degree ranking etc.), while in other cases the initial cooperators are randomly placed (e.g., random ranking) (Fig. 3).

As shown in the Supplement (Figs. S1 and S2), for the harmony game and snowdrift game, the cooperators can fully invade the network easily when the selection strength is strong; on the other hand, for the prisoner’s dilemma, the restoration of cooperation can be only achieved in scale-free networks (Figs. S1 and S2). Therefore, we mostly focus on the stag-hunt game ($S=-0.5$ and $T=0.5$), and identify the ways cooperators can be added in the right places to facilitate the spreading and the restoration of cooperation. For the other games, see the Supplement (Figs. S3, S4 and S5 and S6).

The performance of the different rankings (i.e., their ability to lead to the restoration of cooperation) depends on the structure of the network. Structural rankings are more likely to select high-degree nodes as initial cooperators; intuitively this means that structural rankings facilitate the restoration of cooperation in random and scale-free networks, which have a high diversity in their degree distributions (see Fig. S1 in the Supplement).

In general, the restoration of cooperation increases with the increase of density $\rho$ of initial cooperators, and this is more evident when the selection strength is strong (Fig. 5). For weak selection and in random or scale-free networks, we can find that the performance of the ranking based on N-weighted degree is similar to that of the other structural rankings when the density of initial cooperators $\rho$ is low; however this changes when the density $\rho$ gets higher (Fig. 5 upper panel); this is due to the fact that the initial cooperators selected by the N-weighted degree ranking are more likely to keep far away from the central clusters (Fig. 2). For strong selection, even the random ranking has a good performance in terms of cooperation restoration when the density of initial cooperators $\rho$ is sufficiently large (Fig. 5 upper panel).

Notably, the density of initial cooperators corresponding to high chances of cooperation restoration for the random ranking is different depending on the network structure. In particular, it increases when the network structure is changed from lattice, to small-world, to random network and scale-free network (Fig. 5, upper panel).

We define $\rho _c$ as the critical density, at which the probability of restoration reaches 0.5. Interestingly, the critical density of structural rankings gets lower with the network changing from lattice to scale-free one, especially at strong selection while, in this same scenario, the critical density of the random ranking increases (Fig. 5). The random ranking, however, performs quite good in the lattice network; on the other hand, when the network is scale-free, a larger number of initial cooperators is necessary (Fig. 5b,c).

In the Supplement Fig. S1 we plot an overall comparison of the best performing ranking in various conditions, considering the 6 ranking strategies, varying selection strength, density and network structures.

**Figure 6:**
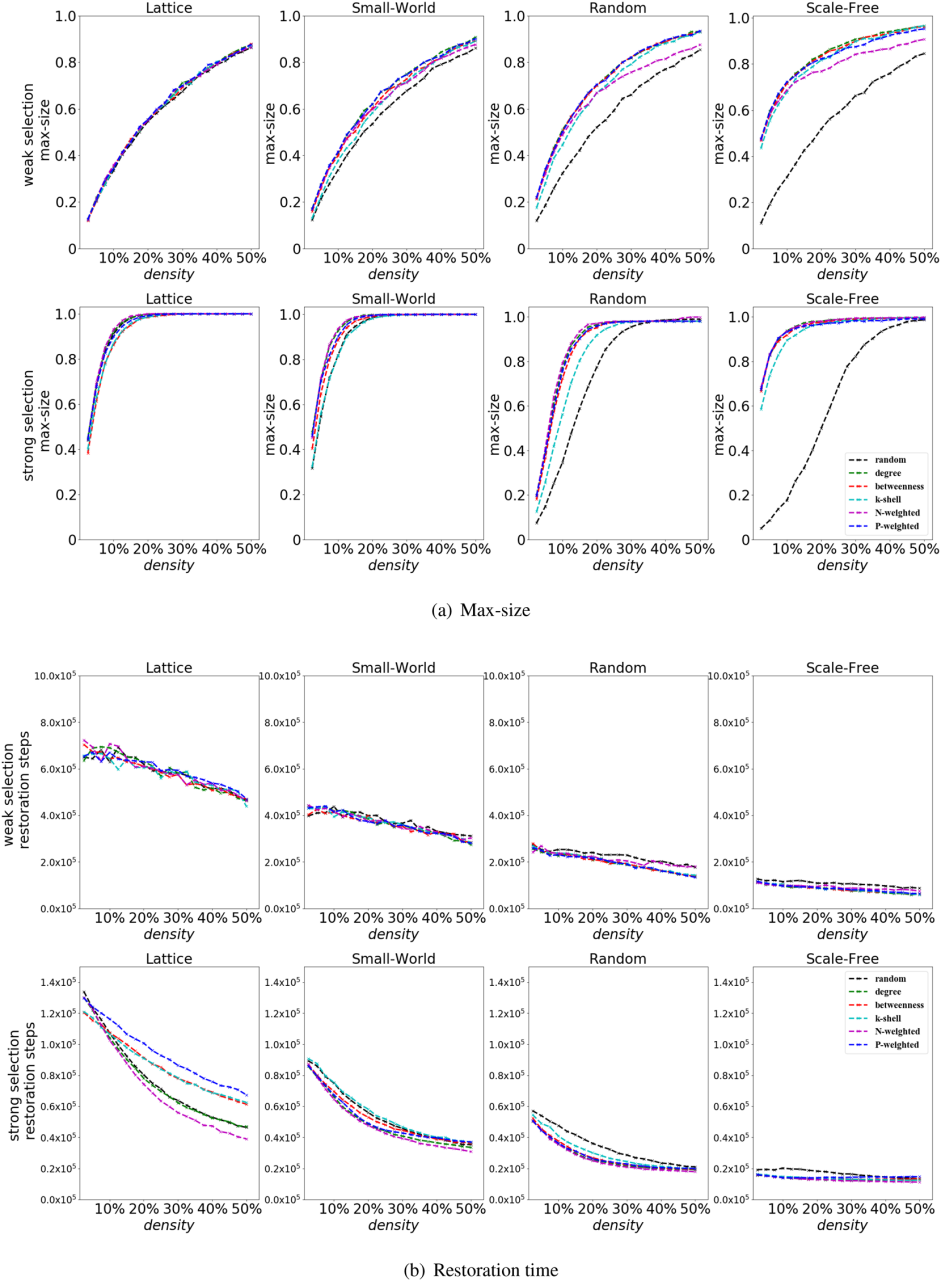
The max-size and restoration time for different initial densities $\rho$. A larger max-size of cooperators and a smaller restoration time (i.e., which corresponds to a faster restoration of cooperation) are obtained by using the N-weighted degree ranking, especially in the lattice and at strong selection. The random ranking works worse (in scale-free networks) than the structural rankings. We consider a stag-hunt game (with $S=-0.5$ and $T=0.5$), and a lattice, small-world, random and scale-free networks, for weak selection ($w=0.001$) and strong selection ($w=0.1$).

Furthermore, we also analyze (Fig. 6) the maximal amount of cooperators reached during the process of restoration of cooperation (i.e., referred as max-size) and the restoration time i.e., average duration (in number of steps) from the introduction of cooperators to the restoration of cooperation. Intuitively this last measure describes how fast cooperation spreads when the restoration is obtained (see Methods for details).

We can notice that, despite similar chances of cooperation restoration, we can find a larger max-size of cooperators and smaller restoration time for the N-weighted degree ranking, especially in the lattice and at strong selection (Fig. 6). On the other hand, in scale-free networks, at weak selection, N-weighted degree and random rankings perform worse than the other rankings (they have a larger restoration time and smaller max-size, see Fig. 6a,b).

Overall, as we can see in Fig. 6, the different positioning of the initial cooperators can make a big difference not only for the chances to obtain the restoration of cooperation but also on how fast the restoration is obtained-in fact, even rankings with similar performance [in terms of probability of cooperation restoration shown in (Fig. 5)] can have very different time of restoration (Fig. 6b). The restoration time obtained using the random ranking is smaller than the one obtained with some structural rankings (e.g. P-weighted degree) in the lattice, even if the random ranking is associated to similar probability of cooperation restoration, indicating that, in some scenarios, we can achieve same chances of cooperation restoration, but faster restoration, by simply adding the initial cooperators in a random manner (Fig. 6b).

### The effects of networks and selections on the restoration of cooperation

The difference of positions of the initial cooperators is caused by the different ranking strategies considered. The performance of the distinct rankings, how easy and fast they allow to recover cooperation, is highly dependent on the games, selections and networks. We present the restoration of cooperation from the perspective of network structures in the supplement Fig. S2.

**Figure 7:**
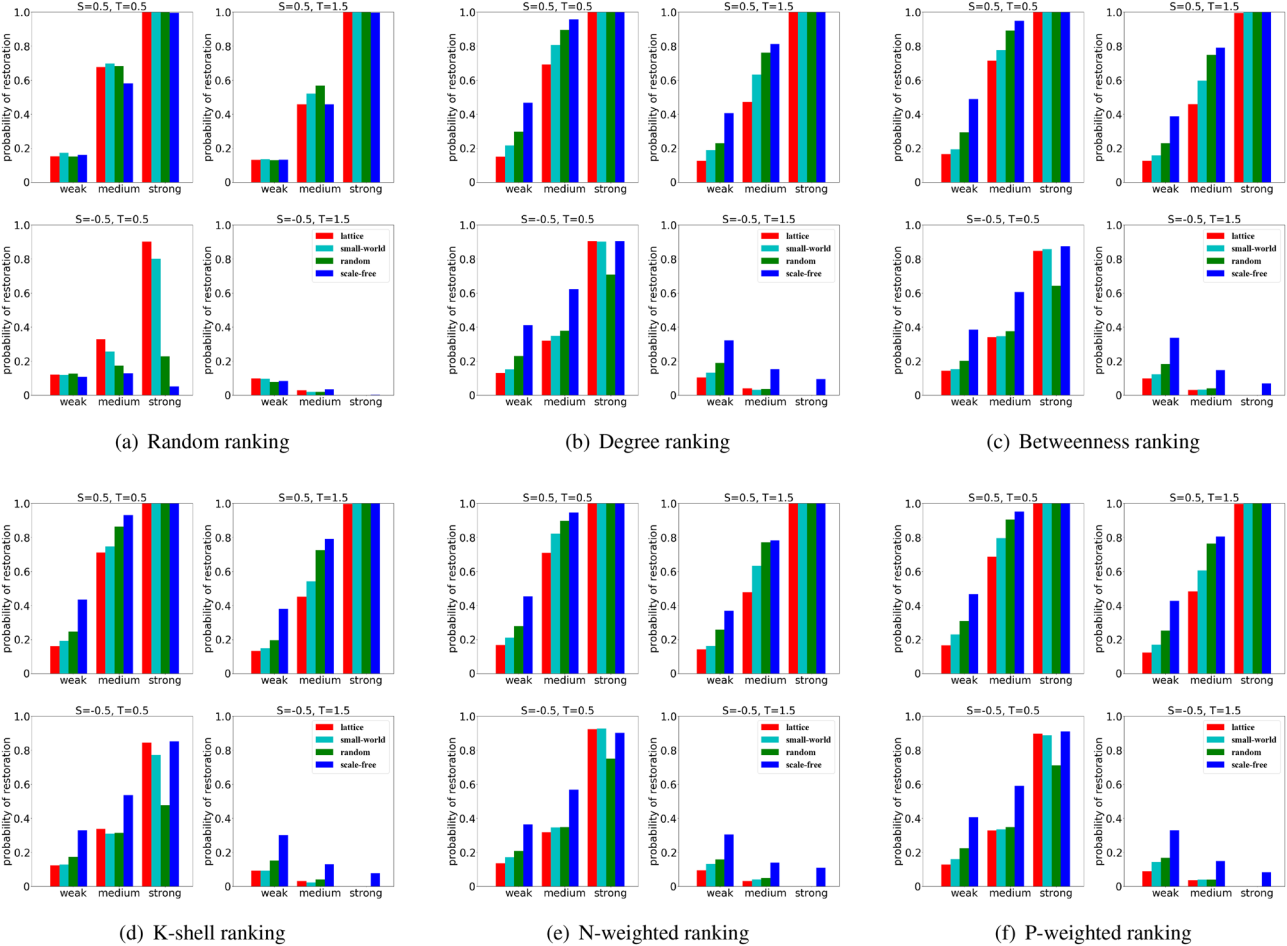
Strong selection weakens the performance of random ranking but enhances the performance of structural rankings in scale-free networks for stag-hunt games. More generally, the increase of degree diversity in the network structure facilitates the restoration of cooperation when the initial cooperators are added using structural rankings. For a fixed initial density $\rho =10\%$, we show the probability of cooperation restoration obtained in a lattice, small-world, random and scale-free networks, at weak selection ($w=0.001$), medium selection ($w=0.01$) and strong selection ($w=0.1$). For completeness we consider all six ranking strategies considered. We explore four types of games: stag-hunt game ($S=-0.5$ and $T=0.5$), prisoner’s dilemma ($S=-0.5$ and $T=1.5$), harmony game ($S=0.5$ and $T=0.5$) and snowdrift game ($S=0.5$ and $T=1.5$).

Moving from a lattice to a small-world network and then to a random network and finally to a scale-free network we can observe that the diversity of the degree is growing (Fig. 3), which generally facilitates the restoration of cooperation for structural rankings. With the increase of degree diversity, the nodes located around the hubs are more likely to be selected under structural rankings, which enhances the advantage of cooperators, as we can observe in the ranking based on degree, betweenness, k-shell, N-weighted degree and P-weighted degree in Fig. 7b–f. However, for random ranking the situation is quite different: scale-free networks don’t improve the chances of cooperation restoration, and such ranking performs worse when used in a scale-free network than in the other types of networks such as lattice or small-world networks, especially when the strength of selection is strong (Fig. 7a).

Furthermore, a stronger selection strength generally amplifies the payoff difference between the individuals. We can observe that for the harmony game, snowdrift game and stag-hunt game, stronger selection facilitates the restoration of cooperation (Fig. 7). For the prisoner’s dilemma, however, stronger selection makes the cheaters more competitive with relative large fitness, and the restoration of cooperation is inhibited at strong selection (the case in Fig. 7 with $S=-0.5$ and $T=1.5$).

Notably, in the stag-hunt game, we can find that the probability of cooperation restoration associated to the random ranking decreases with the increase of the degree diversity of the networks, while the scenario is opposite for the the degree ranking, especially at weak or medium selection (the case in Fig. 7 with $S=-0.5$ and $T=0.5$). When both the temptation to defect and the loss to cooperate are low, i.e., in the case of the stag-hunt game, the high-degree nodes in scale-free networks selected as cooperators are around the hubs and will be more likely to get together and foster the success of cooperation restoration. On the contrary, if the cooperators are selected randomly, they will be less competitive in scale-free networks than in the lattice (the Fig. 7a with $S=-0.5$ and $T=0.5$).

### Structural versus random ranking

**Figure 8:**
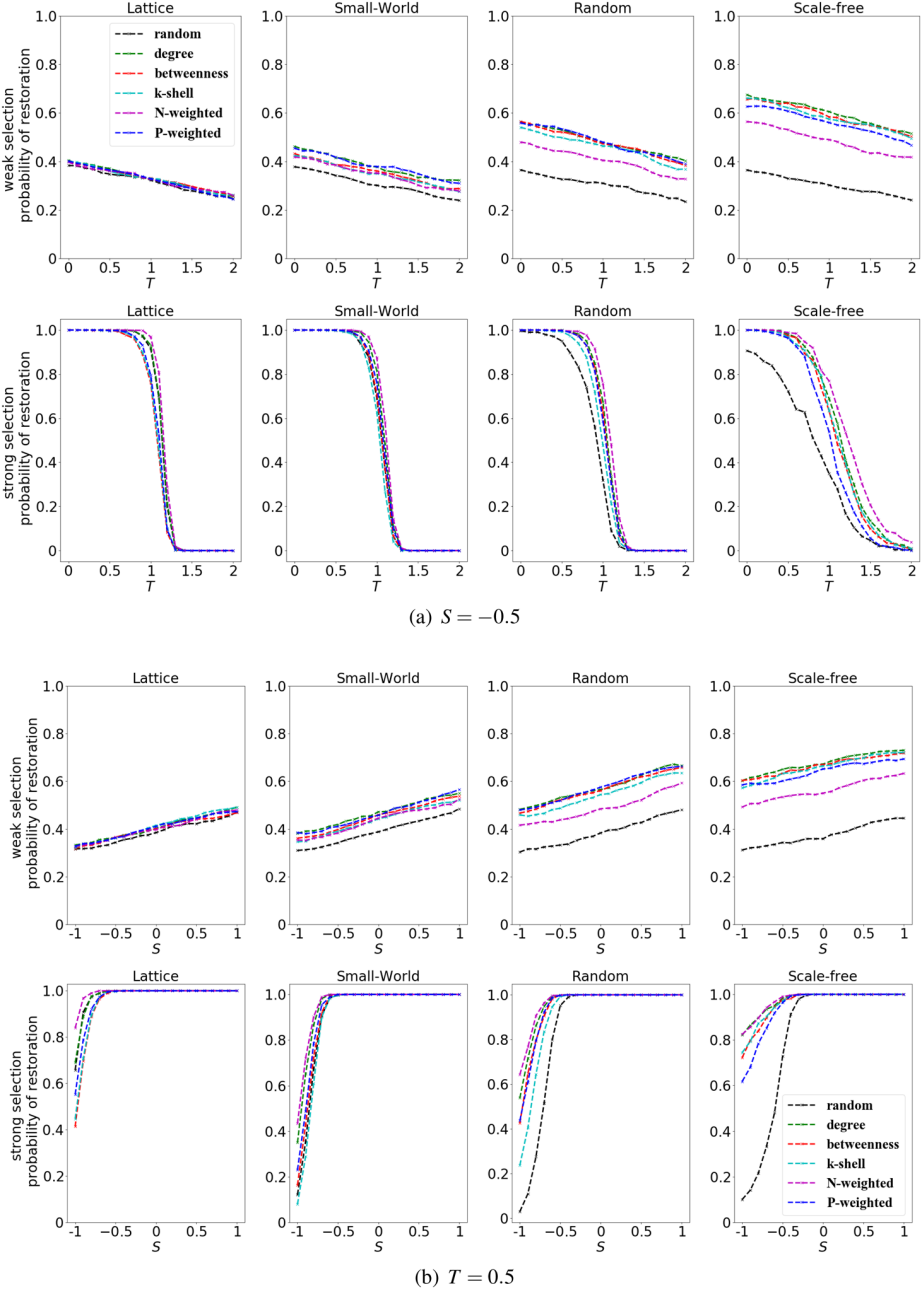
Random ranking works better in lattices than in scale-free networks. We show the probability of cooperation restoration for different networks and rankings. For a fixed $S=-0.5$, the values of *T* are varied between 0 and 2 (upper panel), and for a fixed $T= 0.5$, the values of *S* are varied between − 1 and 1. The initial density of cooperators is $\rho =30\%$, and the probability of cooperation restoration is computed at weak ($w=0.001$) and strong selection($w=0.1$).

To shed light upon the difference of restoration caused by different rankings on different type of networks, we compute the probability of cooperation restoration by fixing *S* and varying *T*, or by fixing *T* and varying *S*. As shown in Fig. 8, for a fixed *S*, the restoration of cooperation is inhibited with the increase of *T*. On the contrary, for a fixed *T*, the restoration of cooperation is enhanced with the increase of *S*. Interestingly, for random ranking, the restoration of cooperation in scale-free networks performs worse than that in lattices; for the other structural rankings, however, the opposite happens - they work better in scale-free networks.

**Figure 9:**
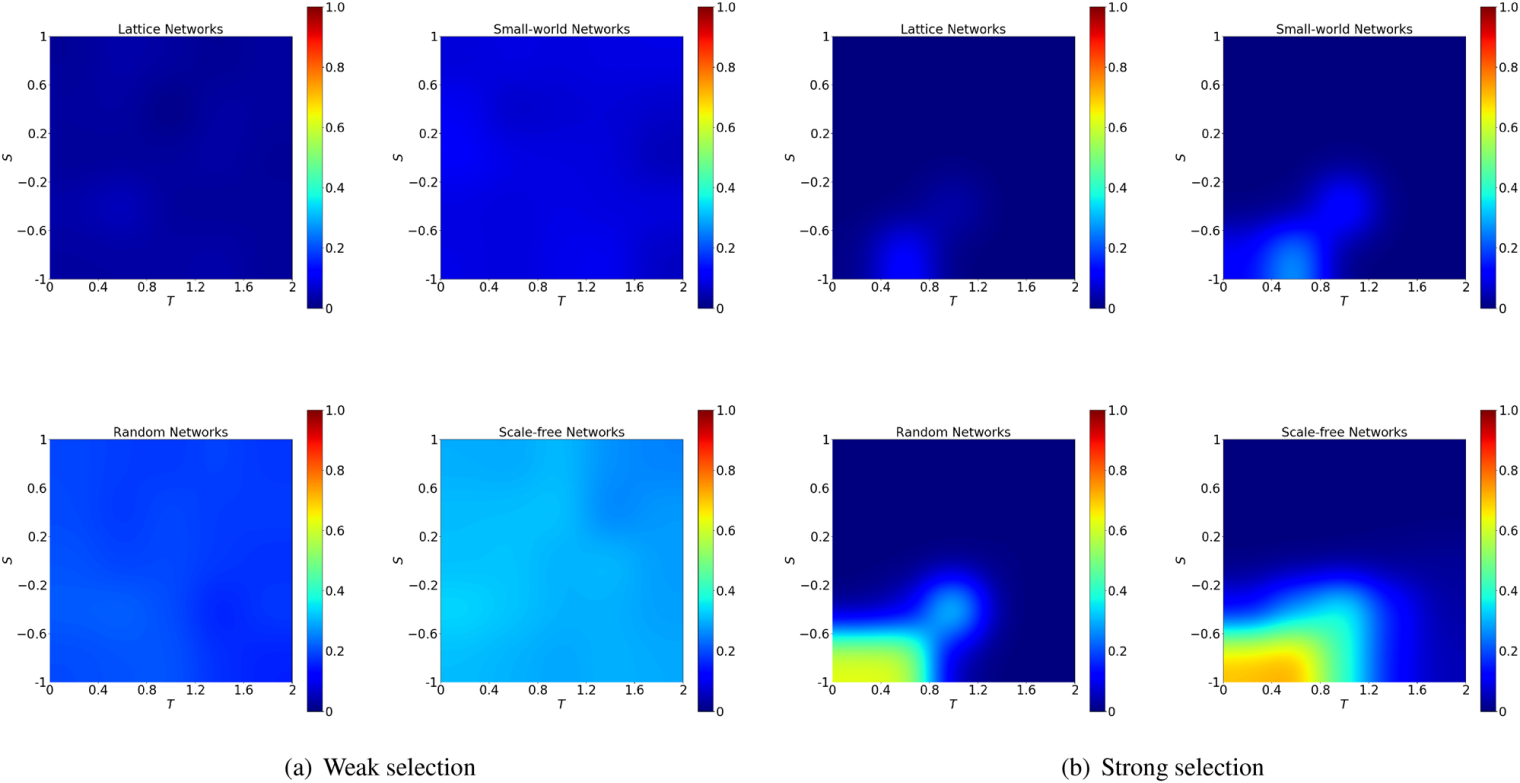
Difference of performance between the best ranking and the random ranking is evident at strong selection for stag-hunt games in scale-free networks. The figures show the difference in the probability of cooperation restoration between the one obtained by using the random ranking and the one obtained using the best structural ranking (i.e., the one which gives the highest probability of cooperation restoration). The biggest gap is obtained in scale-free networks, stag-hunt game and for strong selection. For a fixed initial density $\rho =30\%$, we show the gap between the best performing ranking and the random ranking at weak selection $w=0.001$ (left panels) and strong selection with $w=0.1$ (right panels). The networks considered are lattice, small-world, random and scale-free, respectively. For each subfigure, the x-axis indicates the game parameter $T\in [0,2]$ and the y-axis indicates the game parameter $S\in [-1,1]$.

For a given initial density of cooperators $\rho$, we explore the difference between the best ranking and the random ranking in terms of the probability of cooperation restoration (Fig. 9), for various kinds of networks, at weak selection ($w =0.001$) and strong selection ($w =0.1$).

For a given value of *S* and *T*, the probability of cooperation restoration for random ranking is defined as $p_r$ (similarly, for the other rankings, $p_d$, $p_b$, $p_k$, $p_n$ and $p_p$ are the probability of cooperation restoration for degree ranking, betweenness ranking, k-shell ranking, N-weighted and P-weighted degree ranking, respectively). We define as $p_{best} = \min \{p_r,p_d, p_b, p_k, p_n, p_p\}$ the best ranking. i.e., the one with the maximum value for the probability of cooperation restoration, and the gap of the probabilities of cooperation restoration between the best and the random ranking is computed as $p_{best} -p_r$.

At weak selection, the gap between the best ranking and the random ranking is evident in particular in the scale-free networks (Fig. 9a); however at strong selection, the gap reaches the maximum in scale-free networks around the area of $S \in [-1,0]$ and $T \in [0, 1]$ (Fig. 9b), indicating structural rankings are more effective than random ranking in the case of stag-hunt game.

As we can see in Fig. 9, the difference between structural and random ranking in the probability of cooperation restoration is more evident when the competition is strong.

## Discussion

The restoration of cooperation in a population of cheaters and the collapse of cooperation are two important lines of research in the study of the evolutionary dynamics for structured populations. In this paper we have explored the influence of the initial positioning of cooperators on the success of cooperation restoration. We studied the restoration of cooperation using a large number of numerical simulations, and we show that the structure of the network, the strength of selection, the initial density of cooperators and the ranking strategies play a significant role to identify the best way to position the initial cooperators and maximize the chances of obtaining the restoration of cooperation. Scale-free networks and strong selection can promote the restoration of cooperation in the harmony game, snowdrift game and stag-hunt game, while the restoration of cooperation is generally inhibited in the prisoner’s dilemma. Moreover the way in which the initial cooperators are placed on the network is crucial - the random ranking (i.e., the initial cooperators are placed randomly on a network) in scale-free networks performs worse than in the case of a lattice, small-world or random network, while the opposite holds for structural rankings (i.e., the initial cooperators are placed in strategically - chosen positions). In general, our results show that the restoration of cooperation in structured populations can be obtained using a sort of trade-off: by either adding a large number of cooperators, randomly placed on the network or by adding a much smaller number of initial cooperators but strategically placed in key positions of the networks, using an appropriate structural ranking (Fig. 5). The way the initial cooperators are placed in a network affects not only the chances of observing the restoration of cooperation (Fig. 5) but also the time needed to observe such restoration (Fig. 6). This work then highlights that when studying structured populations, a crucial aspect to consider is the way in which the initial invading cooperators are added into the population-how many cooperative invaders and where they are initially placed in the network can dramatically affect the contagion of cooperation.

## Methods

In the traditional centrality measurements, degree centrality only considers the number of nearest neighbours and betweenness centrality measures the number of connected paths through a nodes. Notably, the *k-shell* of a node is defined in^8^ as a way to measure the location of a node as well as its degree. In k-shell decomposition, all isolated nodes have coreness $c_0=0$, and then all nodes with degree 1 are removed, causing a reduction of the degree values for the remaining nodes. The main idea is to keep removing nodes with degree of 1 until the degree of the remaining nodes is bigger than 1. The coreness of the removed nodes at round 1 is defined to be $c_1 = 1$, while the coreness of the nodes removed at round *t* is defined to be $c_t = t$. A larger coreness for a node means that the corresponding node is located in a more central position (i.e., it has a higher k-shell).

The weighted degree decomposition (WDD) is defined in^25^ as a way to compute the importance of nodes by selecting the nodes one by one as seed nodes. This method ranks the nodes by weighted degree, which is computed by considering the difference between the adjacent seed nodes and the remaining neighbours. The WDD procedure is implemented as:

- the node with the maximum weighted degree is selected as a seed node;
- update the weighted degree for the remaining nodes;
- repeat the above steps until the number of seed nodes reaches *n*.

In detail, the weighted degree of node *v* is computed as:

4 $$\begin{aligned} k^\star (v) = k_p(v) + \Sigma _{u \in NS(v)} (\alpha +\beta |NS(u)|) \end{aligned}$$

where $k_p(v)$ is the number of normal nodes in the neighbourhood, and *NS*(*v*) is the set of neighbours who have been selected as seed nodes. There are two weight coefficients $\alpha$ and $\beta$ to allow a trade-off between exploitation and exploration. Depending on the values of $\alpha$ and $\beta$, we have the N-weighted degree ranking or the P-weighted degree ranking. For the N-weighted degree ranking (namely negative $\alpha$ and $\beta$ coefficients), the seed neighbours as well as their seed neighbours will be under-rated to avoid the overlap of sphere of influence. For the P-weighted degree ranking (namely positive $\alpha$ and $\beta$ coefficients), however, its seed neighbours as well as their seed neighbours will be over-rated in order to exploit the advantages of hubs.

We measure the performance of the different rankings by considering:

- the probability of cooperation restoration, which is calculated by the ratio between the number of simulations leading to the restoration of cooperation and the total number of simulations.
- the max-size of restoration, which is the maximal number of cooperators recorded after the initial addition of cooperation (it is the size *N* of the population if there is the restoration of cooperation).
- the restoration time, which is the number of update steps the system takes to reach the full cooperation restoration, after the initial addition of cooperators.

## Supplementary Information

Supplementary Information.
