# Supplementary material for: Strategically positioning cooperators can facilitate the contagion of cooperation
Source: Sci Rep. 2021 Jan 13;11:1127. doi: 10.1038/s41598-020-80770-8 (PMC7806618; doi:10.1038/s41598-020-80770-8)
Supplement: Supplementary file 1 — Supplementary Information. [file 41598_2020_80770_MOESM1_ESM.pdf]

Strategically positioning cooperators can facilitate the contagion of cooperation
Supplementary Material

Guoli Yang, Matteo Cavaliere, Cheng Zhu, Matjaž Perc

1 Performance of the rankings

In order to obtain the ranking with the best performance (in terms of cooperation restoration) in various conditions, we systematically compare the 6 ranking strategies introduced in the main text from the perspective of selections, density and networks (Figure S1). We consider 4 classical games: the harmony game ($S = 0.5$, $T = 0.5$), the snowdrift game ($S = 0.5$, $T = 1.5$), the stag-hunt game ($S = -0.5$, $T = 0.5$) and the prisoner's dilemma ($S = -0.5$, $T = 1.5$). At weak selection, the restoration of cooperation is enhanced in networks with high degree diversity, especially in scale-free networks, where the high-degree nodes will be more likely selected as cooperators when structural rankings are used (such as degree ranking, P-weighted degree ranking). When the selection strength is increased, and the initial density is low, all the considered ranking strategies facilitate the restoration of cooperation in the lattices; however the random ranking performs increasingly worse as the network degree diversity increases (Figure S1(b)). With the increase of initial density, a complete restoration of cooperation can be reached in the harmony, stag-hunt and snowdrift games when the selection strength is strong. However, in the prisoner's dilemma, strong selection inhibits the restoration of cooperation due to the relative high fitness of cheaters and the high resilience of cheating communities (Figure S1(d)). It should be noted that the probability of cooperation restoration in scale-free networks is higher than the corresponding in the lattice, small-world and random networks, which reveals that the heterogeneous structure of networks can help the formation of cooperative clusters in the restoration of cooperation (in particular when coupled to a strategical way, i.e., ranking, to add the initial cooperators, Figure S1).

Moreover, we can observe that the performance (in terms of cooperation restoration) of the N-weighted degree ranking at strong selection and high initial density of cooperators outperforms the other types of rankings in the prisoner's dilemma, which is caused by the strategic positioning of initial cooperators. Intuitively, for that ranking, the cooperators will be positioned away from the hubs which will help them to avoid connections to the cheaters usually positioned around the hubs of the network.

2 The effects of the network structure

Moving from a lattice to a small-world network and then to a random network and finally to a scale-free network we can observe that the diversity of the degree is growing, which generally facilitate the restoration of cooperation for structural rankings. With the increase of degree diversity, the nodes located around the hubs are more likely to be selected under the structural rankings, which enhances the advantage of cooperators (for example the ranking of degree in Figure 2(b)). However, for random ranking the situation is different: scale-free networks don't improve the chances of cooperation restoration, and such ranking perform worse when used in a scale-free network than in the other types of networks such as lattice or small-world networks, especially when the strength of selection is strong (Figure 2(a)).

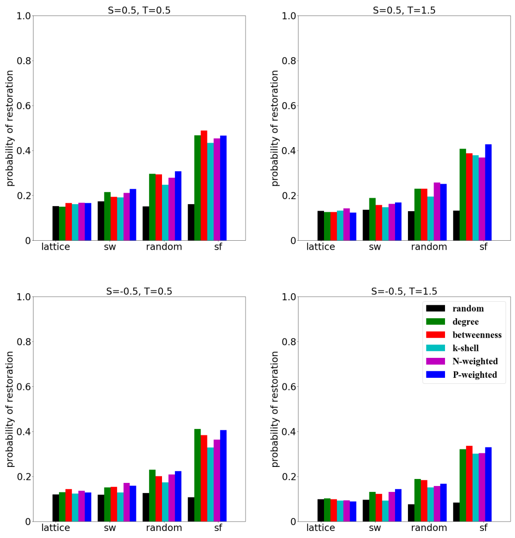

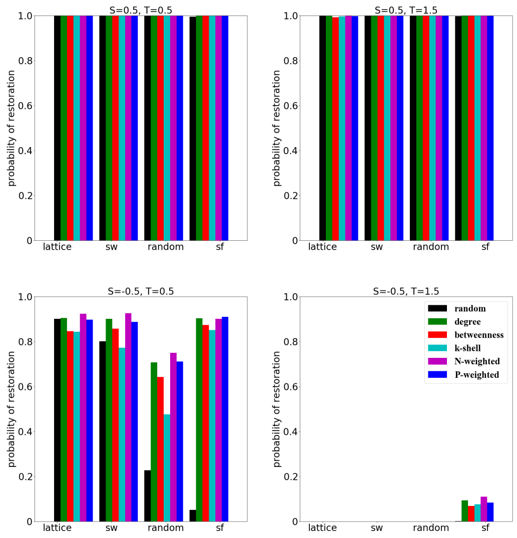

(a) Weak selection, Low density

(b) Strong selection, Low density

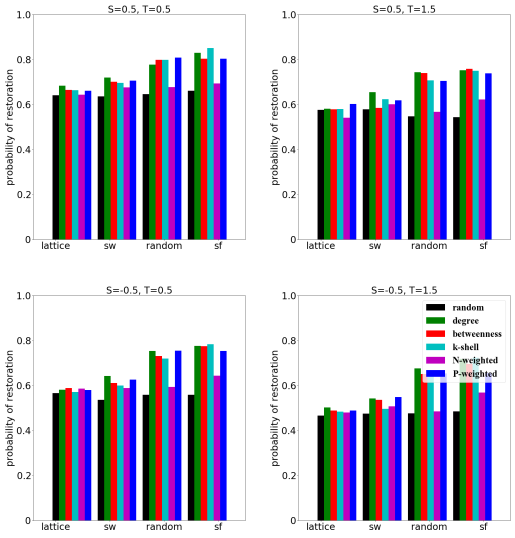

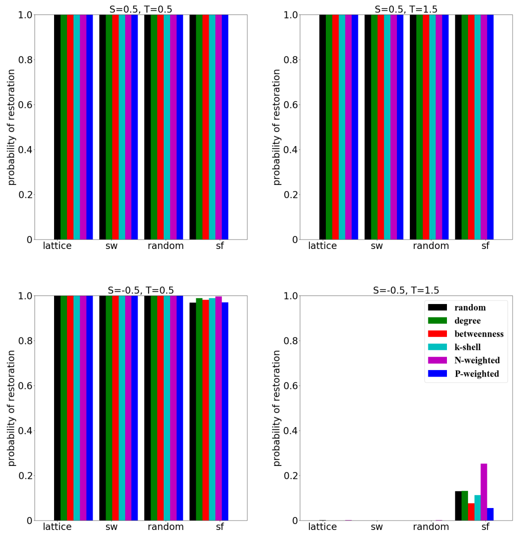

(c) Weak selection, High density

(d) Strong selection, High density

Figure S1: The chances of cooperation restoration depends on the selection strength, initial density, rankings and network structure. With initial density of $\rho = 10\%$ (upper panels) and $\rho = 50\%$ (lower panels), the probability of cooperation restoration is shown for $w = 0.001$ (left panel) and $w = 0.1$ (right panel). For each subfigure, the x-axis indicates the type of networks (lattice, small-world, random and scale-free), and the y-axis indicates the probability of cooperation restoration. We consider the random and structural rankings, and different values of S and T which correspond to different games.

3 The effects of the initial density

As we have presented the restoration of cooperation for the game of stag-hunt in the main texts, here we will study the effects of the initial density of cooperators on the restoration of cooperation for the harmony game, snowdrift game and prisoner's dilemma.

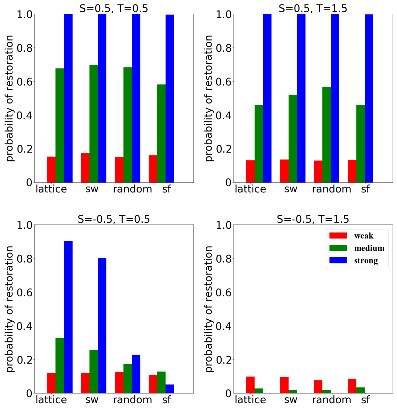

(a) Random ranking

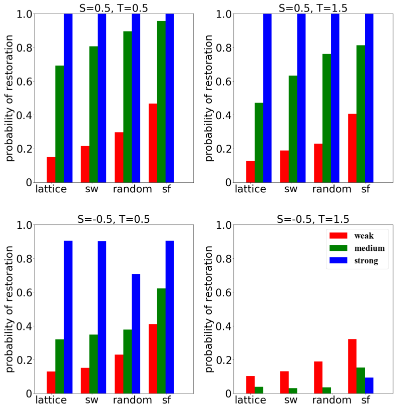

(b) Degree ranking

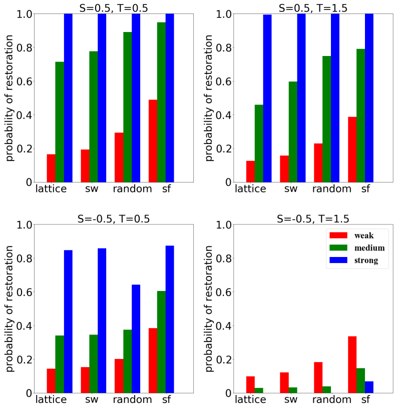

(c) Betweenness ranking

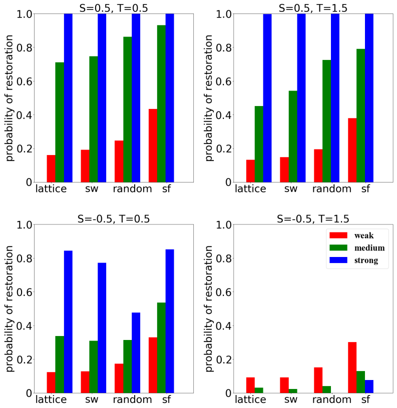

(d) K-shell ranking

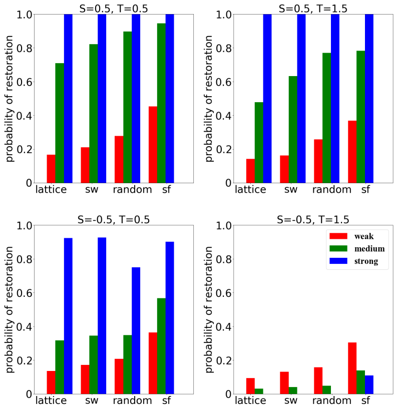

(e) N-weighted ranking

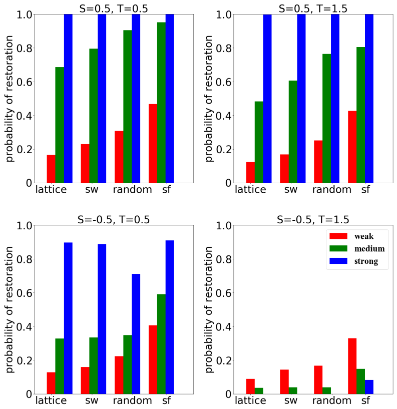

(f) P-weighted ranking

Figure 2: Scale-free networks inhibit the performance of random ranking for stag-hunt games. For a fixed initial density $\rho = 10\%$, we plot the probability of cooperation restoration obtained for a lattice, small-world (sw for short), random and scale-free (sf for short) networks at weak selection ($w = 0.001$), medium selection ($w = 0.01$) and strong selection ($w = 0.1$). Six ranking strategies are considered: the random ranking, the degree ranking, the betweenness ranking, the k-shell ranking, the N-weighted degree ranking and the P-weighted degree ranking. Four types of games are explored, including stag-hunt game ($S = -0.5$ and $T = 0.5$), prisoner's dilemma ($S = -0.5$ and $T = 1.5$), harmony game ($S = 0.5$ and $T = 0.5$) and snowdrift game ($S = 0.5$ and $T = 1.5$).

3.1 Harmony game

We consider a harmony game (with $S = 0.5$ and $T = 0.5$) and present the probability of cooperation restoration, the max-size of cooperators and the average duration from the introduction of the initial

cooperators to the end of cooperation restoration (restoration time), Figure S3.

The increasing initial density of cooperators ρ fosters the success of restoration at weak selection, but fails to do that at strong selection (Figure S3).

3.2 Snowdrift game

Here we consider a snowdrift game (with $S = 0.5$ and $T = 1.5$), and present the probability of cooperation restoration, the max-size of cooperators and the average duration from the introduction of cooperators to the end of restoration (restoration time) in Figure S4. The increasing density ρ fosters the cooperation restoration at weak selection, but fails to do that at strong selection.

3.3 Prisoner's dilemma

We consider the prisoner's dilemma (with $S = -0.5$ and $T = 1.5$) and present the probability of cooperation restoration, the max-size of cooperators and the average duration from the introduction of cooperators to the end of restoration (restoration time) in Figure S5. As we can see, the restoration of cooperation is inhibited at strong selection, especially when the network structure is uniform (i.e., small degree diversity), as in the case of lattice, small-world networks and random networks.

Increasing the initial density of cooperators leads to high chances of the restoration of cooperation at strong selection, but that can only be achieved when the initial density of cooperators reaches very high values. We can also observe that in scale-free networks the ranking based on N-weighted degree outperforms the other rankings, at intermediate initial density of cooperators (Figure S6).

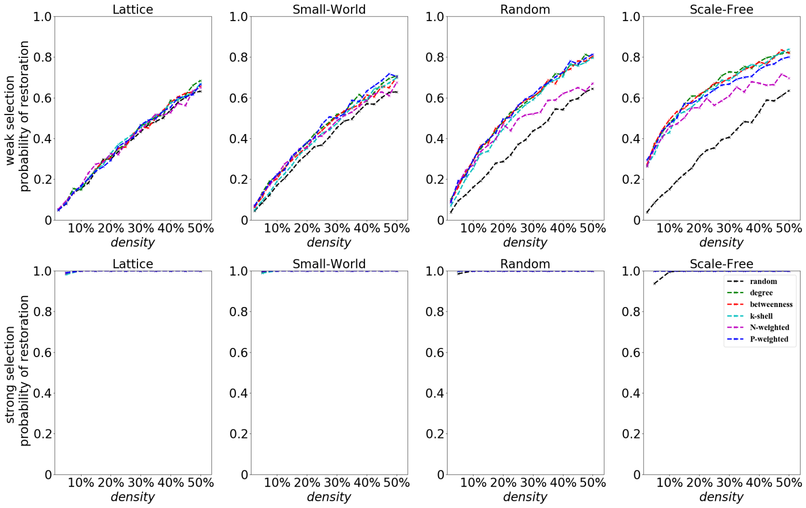

(a) Probability of Restoration

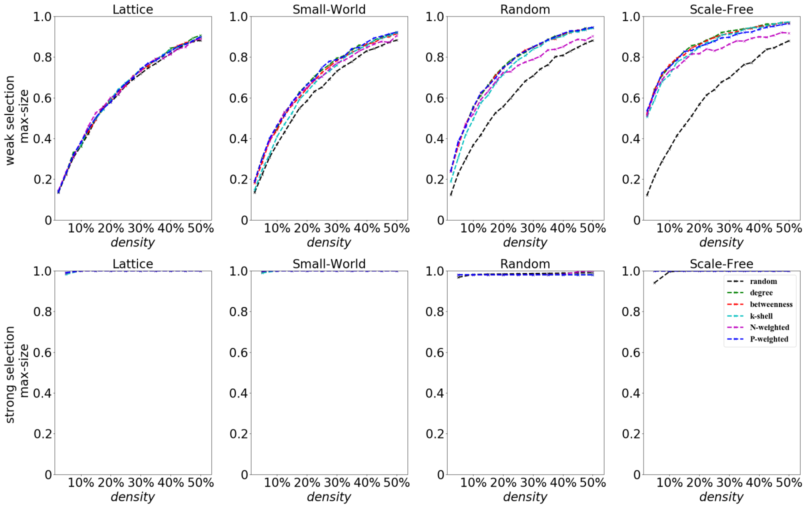

(b) Max-size

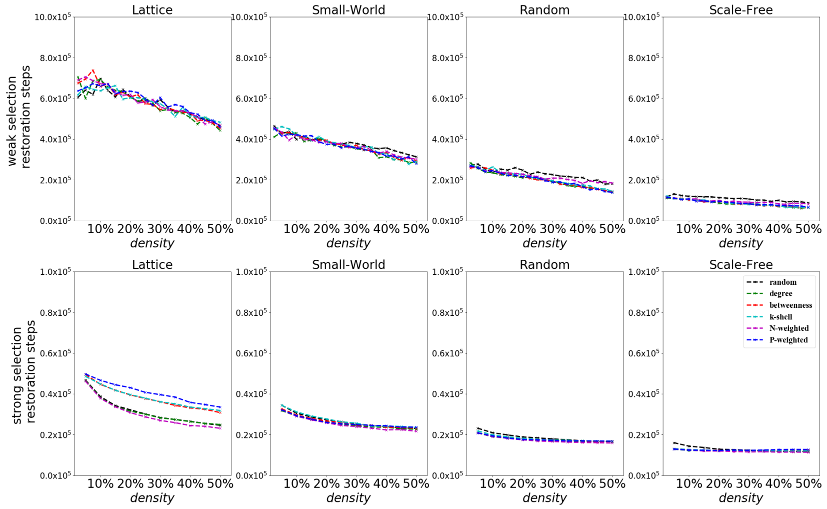

(c) Restoration time

Figure S3: The probability of cooperation restoration is inhibited at strong selection for the harmony game. We show, as function of the initial cooperators density, the probability of cooperation restoration, max-size of cooperation and restoration time for the harmony game (with $S = 0.5$ and $T = 0.5$), in the lattice, small-world, random and scale-free networks, at weak selection ($w = 0.001$) and strong selection ($w = 0.1$).

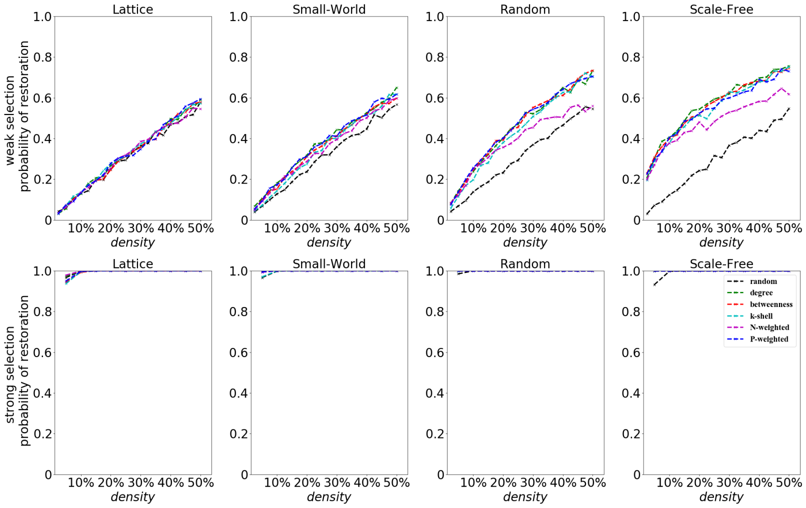

(a) Probability of Restoration

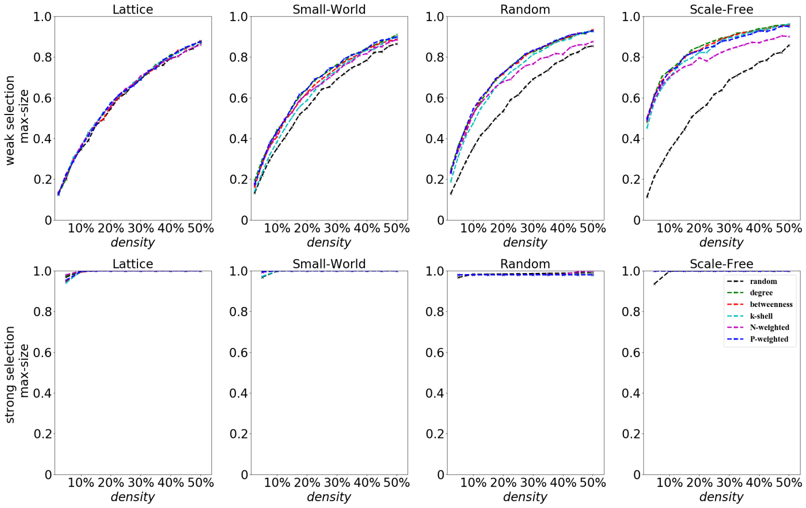

(b) Max-size

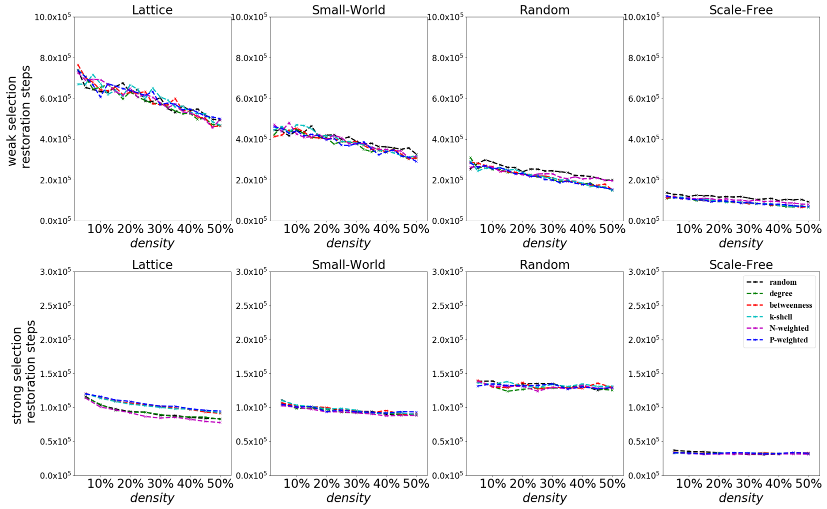

(c) Restoration time

Figure S4: The probability of cooperation restoration is inhibited at strong selection for the snowdrift game ($S = 0.5$ and $T = 1.5$). We show the probability of cooperation restoration, as function of initial cooperators density, for a lattice, small-world, random and scale-free networks, at weak selection ($w = 0.001$) strong selection ($w = 0.1$).

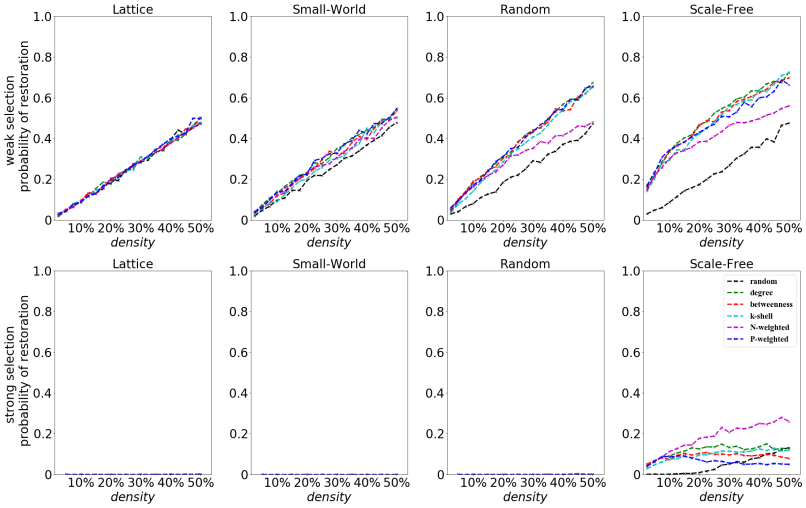

(a) Probability of Restoration

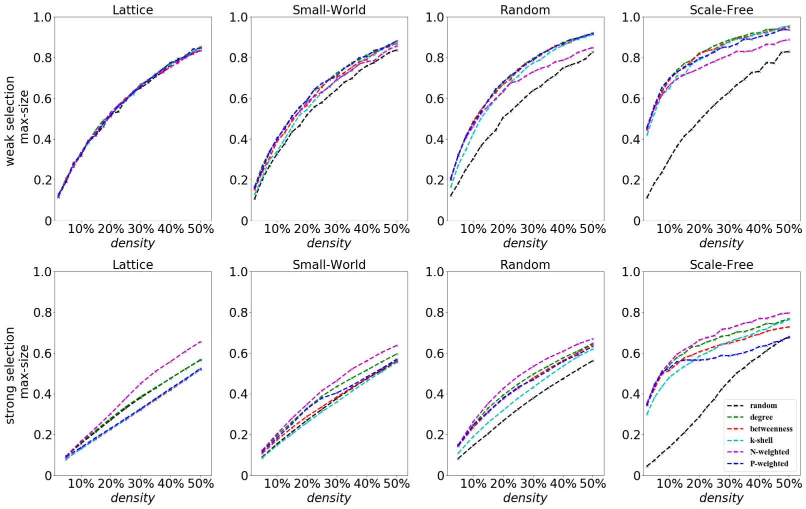

(b) Max-size

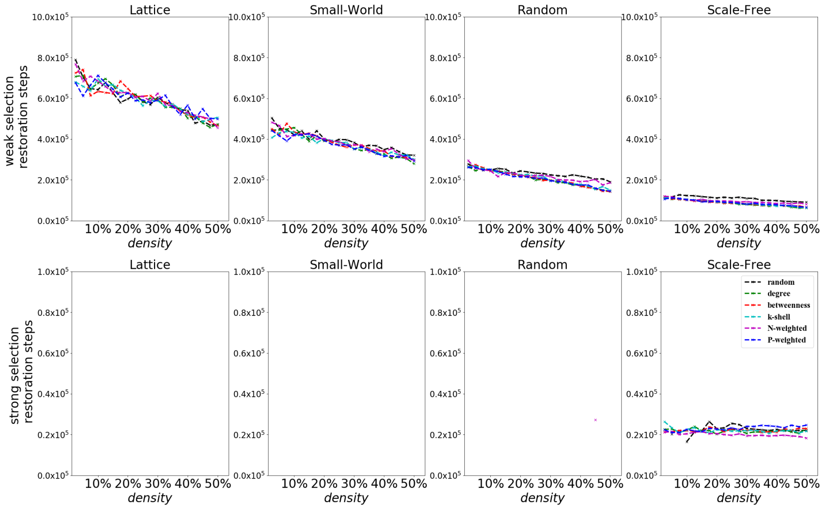

(c) Restoration time

Figure S5: Scale-free networks help the restoration of cooperation at strong selection. For a given Prisoner's dilemma with $S = -0.5$ and $T = 1.5$, we plot the probability of cooperation restoration, the max-size and the restoration time, as function of the initial cooperators density, obtained in the lattice, small-world, random and scale-free networks, at weak selection ($w = 0.001$) strong selection ($w = 0.1$).

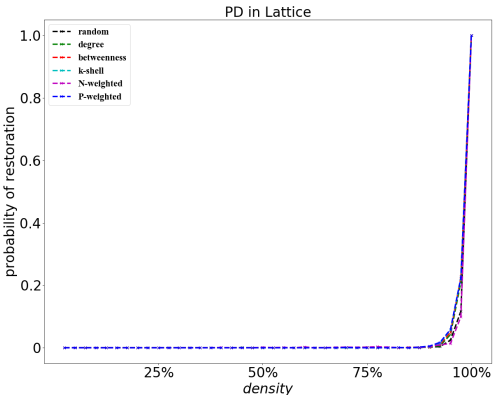

(a)

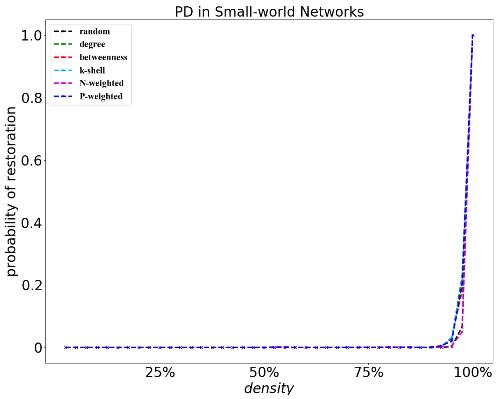

(b)

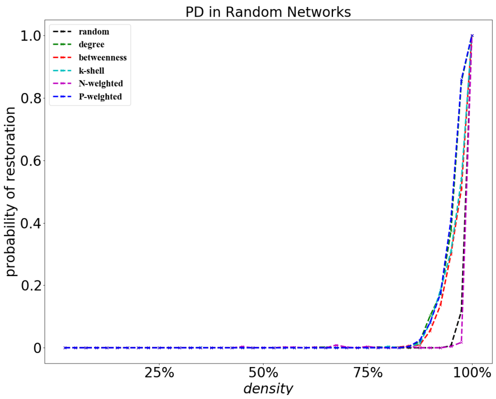

(c)

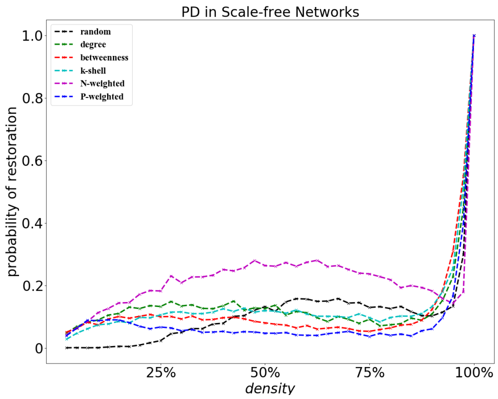

(d)

Figure S6: The probability of cooperation restoration in the prisoner's dilemma increases as the initial density of cooperators is increased. For the prisoner's dilemma (with $S = -0.5$ and $T = 1.5$) we plot the probability of cooperation restoration, as function of the initial cooperators density, for the lattice, small-world, random and scale-free networks at strong selection ($w = 0.1$). In scale-free networks (bottom-right panel) the N-weighted degree ranking outperforms the other rankings, at intermediate initial density of cooperators.
